# Involving older people in research: practical considerations when using the authenticity criteria in constructivist inquiry

**DOI:** 10.1111/j.1365-2648.2010.05500.x

**Published:** 2011-03

**Authors:** Christine Brown Wilson, Philip Clissett

**Affiliations:** Christine Brown Wilson PhD RN Lecturer School of Nursing, Midwifery and Social Work, University of ManchesterUK; Philip Clissett PhD MA RN Lecturer School of Nursing, Midwifery and Physiotherapy, University of NottinghamUK

**Keywords:** constructivism, older people, users and carers, users involvement in research

## Abstract

**Aim:**

The purpose of this paper is to identify practical suggestions that could enable other researchers to consider how quality may be evidenced using constructivist principles including the perspectives of older people and their caregivers.

**Background:**

Constructivism suggests that reality is part of a social construction, which holds different meanings for each person, in which people are active agents, making autonomous decisions. This approach to research has been identified as suitable for health and social care professionals because these underpinning principles reflect the values of these professions, facilitating the involvement of users and carers. The authenticity criteria have been developed to reflect these philosophical principles but have been criticized for their inaccessible language. To incorporate user and carer perspectives, the criteria have been revised into a more accessible model matrix known as the AldreVast Sjuharad criteria.

**Discussion:**

This paper reports on two constructivist studies that explored relationships between older people, families and staff in different settings – the community and care homes. Examples from both settings demonstrate how the perspectives of users and carers were incorporated throughout the research process. Following the AldreVast Sjuharad model matrix, practical guidance is provided on how the quality of constructivist research may be implemented in nursing research.

**Conclusions:**

The different settings in this paper influenced how the AldreVast Sjuharad model matrix was applied. Further work is needed in exploring how the perspective of users and carers may be incorporated into the quality process of constructivist research.

## Introduction

In the helping professions, there is a move away from traditional views of expertise to a recognition and respect for the expertise and knowledge of users and carers (Reed *et al.* 2004). This has resulted in a widespread acceptance of the principles of user and carer involvement in research, although the criteria used to judge the success of such initiatives remains less well developed (Bradbury & Reason 2001). Constructivism is a relatively new addition to the field of qualitative research in nursing and has been described as having an inclusive approach, facilitating the involvement of users and carers throughout the research process (Hanson *et al.* 2006). It has been identified as ideal for health and social care professionals because the underpinning principles reflect the values of these professions (Rodwell 1998). However with a few exceptions, such as Appleton (1997), Davies and Nolan (2003), Clarke *et al.* (2009), relatively little has been written about constructivism in the context of nursing research.

This paper will give a brief overview of the key principles of constructivist research before exploring how this approach was adopted in two studies that focussed on relationships between older people, their families and staff.

The main issue for consideration will be to explore how the quality criteria identified by Guba and Lincoln (1989), applied in a social work context (Rodwell 1998) and subsequently developed at the AldreVast Sjuharad (AVS) Research Centre in Sweden (Nolan *et al.* 2003, Hanson *et al.* 2006), were applied and evaluated in these studies. The purpose of this paper is to identify practical suggestions that might enable other researchers to consider how quality may be evidenced using constructivist principles while incorporating user and carer perspectives.

## Constructivist research

The constructivist paradigm asserts that perceptions of reality are located in time and place, and are constructed by the individual or individuals (Guba & Lincoln 1989). As a result, constructivist research seeks to generate the most sophisticated description or explanation of a particular setting as a result of an interactive process between the researcher and participants, many of whom are likely to hold differing perspectives about individual situations (Guba & Lincoln 1989). The principles associated with the constructivist paradigm are described in Table 1.

**Table 1:** Principles associated with the constructivist paradigm

Area of concern	Constructivist principle
The nature of reality	Multiple social realities exist. Reality is represented by the most sophisticated and informed construction that can be agreed upon at a particular time
The relationship of the knower to the known	The interaction of the researcher and participant creates knowledge and understanding of the phenomenon under consideration
The possibility of generalization	The concept of generalization is replaced with the notion of tentative application of findings to other, similar settings
The possibility of causal linkages	Due to mutual simultaneous shaping, it is not possible to separate cause from effect
The role of values	Values permeate constructivist research

Adapted from Guba and Lincoln (1989).

Constructivist inquiry arose as a result of dissatisfaction with conventional methods of evaluation as these tended to exclude different stakeholders (Lincoln 2001). This resulted in a shift in the distribution of power through information sharing with and between stakeholders, designing interventions or activities as directed by stakeholders and creating conditions which foster taking action on the outcomes of the inquiry or evaluation (Lincoln 2001). However, although an underpinning value in constructivist inquiry has been to enable the voices of all stakeholders to be heard, criticisms have been directed at the exclusive language used in constructivism preventing user involvement at all levels of the research process (Nolan *et al.* 2003).

This point is particularly relevant with regard to assessing quality in constructivist research. Two sets of criteria have been developed: trustworthiness (Guba & Lincoln 1989– see Table 2) and authenticity (Lincoln & Guba 1985– see Table 3). For a research study to be consistent with constructivist principles and demonstrate that it is worthy of attention, both the trustworthiness and authenticity criteria should be used in the same study (Erlandson *et al.* 1993). While both criteria were used in the studies presented in the following section, this paper will focus on the application of the authenticity criteria, exploring how each criterion was addressed in differing contexts, involving older people, families caregivers and staff.

**Table 2:** Trustworthiness criteria (based on Rodwell 1998)

Aspect of trustworthiness		Interventions undertaken in both studies
Credibility – equated with external validity	The process of understanding the depth and scope of the issues under investigation	Prolonged engagement Persistent observation Peer debriefing Member checks
Dependability – equated with internal validity	The appropriateness of methodological decisions is demonstrated	Purposive sampling ‘Thick’ description
Confirmability – equated with reliability	The results that are reported are linked to the data	Maintenance of a methodological log
Transferability – equated with generalizability	Information created and lessons learned in one environment may be of relevance to other environments. This decision lays with the reader of the report	External review of the development of data into findings and conclusions

**Table 3:** A comparison of the terms used by Guba and Lincoln (1989) and Nolan *et al.* (2003) in relation to the authenticity criteria

Term used by Guba and Lincoln (1989)	Term used by Nolan *et al.* (2003)	Definition of term
Fairness	Equal access	All viewpoints are represented even-handedly
Ontological authenticity	Enhanced awareness of the position of self	Participants understand their situation in more informed ways as a result of participation in the research
Educative authenticity	Enhanced awareness of the position of others	Participants understand the situations of others in more informed ways as a result of participation in the research
Catalytic authenticity	Encouraging action by providing a rationale or impetus for change	Participants have a greater insight into actions that they might take to change their situation as a result of participation in the research
Tactical authenticity	Encouraging action by providing the means to achieve change	Participants feel empowered and enabled to act as a result of participation in the research

Source: Nolan *et al.* (2003).

The authenticity criteria have been described as potentially the most radical dimension of constructivism but there remains only limited guidance as to how the authenticity criteria might be achieved (Rodwell 1998). However, a key issue is language and terminology. If an underpinning assumption of constructivist inquiry is to enable previously marginalized stakeholders to be part of producing the knowledge in the inquiry (Lincoln 2001), then the language should be accessible enabling a full involvement of stakeholders such as vulnerable older people and their carers in all stages of the research process (Nolan *et al.* 2003). Nolan *et al.* (2003) have considered this in relation to the underlying principles of the Authenticity Criteria and developed a more accessible terminology rendering the criteria more comprehensible for all participants involved in the research, promoting the potential for full user participation at all points in the research process (Table 3).

A further development in the search for practical actions to enhance the authenticity of a constructivist study is the development of a matrix identifying how each aspect of the criteria may be applied in the planning, process and product phases of the research (Table 4). This matrix has subsequently been applied in the evaluation of work undertaken by AVS Research Centre, labelled as the AVS model matrix (Hanson *et al.* 2006). However, there remains a dearth of literature giving details about how researchers might use the AVS model to promote the authenticity of constructivist research. Therefore, this paper will discuss how this model was applied in two studies.

**Table 4:** The AVS model matrix (Hanson*et al.* 2006, Nolan *et al.* 2003)

	Planning	Process	Product
Equal access			
Enhanced awareness of self			
Enhanced awareness of others			
Encouraging action			
Enabling action			

## Implementing the authenticity criteria

### The studies

The two studies described in this paper explored relationships between older people, their families and health and social care staff in different contexts: care homes (Brown Wilson 2007) (Study 1) and in the community (Clissett 2007) (Study 2). Both studies used a constructivist approach and are outlined briefly in Boxes 1 and 2.

**Box 1 Study 1: The care home based study (Brown Wilson 2007)**

Using a constructivist framework, three care homes were purposively chosen as case studies. Each care home was chosen as an individual case with defined boundaries and a pattern of behaviour, which would enable the exploration of the range of relationships between residents, families and staff. The choice of three care homes was intended to capture a breadth of experience of residents, families and staff working in different types of home. Data were collected over a 2-year period using participant observation (*n* = 256 hours); interviews with residents (*n* = 10), families (*n* = 18) and staff (*n* = 25) and focus groups (*n* = 7). The researcher spent between four and nine months in each home, over a consecutive period of time. Each home was visited on different days at different times.

Three types of relationships were identified from the data: pragmatic, resident centred and relationship centred. The type of relationships able to be developed in each home was primarily influenced by the approach staff adopted during the process of care delivery. The findings clustered around three methods of care delivery, described as individualized task centred, resident centred and relationship centred. These approaches were not mutually exclusive and some staff were observed to adopt different approaches at different times.

With further consideration of the data across the three care homes, other factors that influenced the type of relationships able to be developed included leadership, motivation and consistency of staff and the contribution of residents and families.

**Box 2 Study 2: The community based study (Clissett 2007)**

This study sought to gain an understanding of caregiving relationships from multiple perspectives over the period of one year. Using a longitudinal approach involving three phases, data were collected during 74 semi-structured interviews relating to 19 caregiving situations. In phase one, these interviews involved 14 community dwelling older people, 17 family carers and 7 professional carers. In phase two, 11 community dwelling older people, 14 family carers and 5 professional carers were involved. Nine community dwelling older people, 12 family carers and 6 professional carers took part in the final phase.

Data analysis revealed four broad types of relationship between the older person and their family carer: actively reciprocal, passively reciprocal, actively discordant and passively discordant. The nature of these relationships appeared to be influenced by the interaction of five underpinning processes: (re)discovering each other, (re)negotiation, recognizing the value of each other, recognizing the difference you are making and (re)discovering pleasure. These five processes also applied to the experiences and contributions of the professional carers.

When considering the data gathered over the period of the year, three broad factors were found to influence changes in the nature of the relationships: responses to difficulties and tensions, changes in the condition of the care recipient, and the impact of professional services. In particular, professional carers tended to have an influence on decision making between the family carer and care recipient and in their ability to (re)discover pleasure.

### Equal access

Equal access reflects the position that all stakeholders should have the opportunity of sharing their perspectives in the research process (Nolan *et al.* 2003). In Study 1, attempts were made in each care home to ensure that the voices of all stakeholder groups were heard by extending a verbal and written invitation to all residents, staff and families to take part in the study. This invitation to participate was reiterated on each visit to ensure negotiated consent. People with hearing, sight, speech or cognitive impairment were also supported to take part if they wished to, which involved a level of flexibility in the research design.

For example, interviews with residents experiencing difficulties with hearing were conducted on days identified by the residents when their hearing was at its optimum. Involving residents with cognitive frailty was achieved through structuring the questions around familiar concepts with the use of pictures to illustrate these concepts.

The key challenge relating to equal access in both studies was that of ensuring that the voice of the care recipient was heard. On the whole, family and professional carers were able to express their perspectives clearly and with ease. However, in Study 2, there were numerous barriers that made this difficult for some of the care recipients: some had speech difficulties that made their words difficult to understand; others spoke very quietly which meant that, while they could be understood, it was never clear whether or not their words would be audible on the tape recording; and others appeared to struggle to concentrate for the length of the interview.

While none of the difficulties in either study could be overcome completely, attempts were made to minimize their impact. For example, when people spoke very quietly, frequent summaries of what the person was saying were offered, with attempts being made to use the exact words of the individual for the points that appeared to be the most pertinent.

### An enhanced awareness of the position of self

The enhanced awareness of the position of self refers to the expectation that, as a result of participating in a constructivist study, the participants should understand their situation, or the situation of the group to which they belong, in a more informed and sophisticated way (Nolan *et al.* 2003).

In Study 1, the participant observation was used to identify examples that might cause participants to re-assess their experience as the same or different to others. These examples were used in the interviews to encourage participants to think of what they did and what they considered to be important in their daily experience as reflected in the following conversation with a group of residents:

I: so do you see yourselves as part of the team?

W: we are yes, we are really

G: yes I think so

I: how do you see yourselves as fitting into being part of the team?

B: we help them a lot

I: so tell me how you think you do help them

B: doing what you’re told

G: well we don’t ring a lot when they are busy

For other residents, interviews and conversations provided opportunities for them to reflect on their lives, speaking of significant issues both in the past and present. For one resident, this seemed to provide a renewed self-awareness as she identified that she had forgotten how interesting her life had been.

Interviews with staff helped them to realize the significance of ‘the everyday’ in their practice. Many of the staff showed evidence of reflexivity in this process and could be described as being aware of the role they played in developing relationships:

I saw she subscribed to the RSPB and asked her about it. She told me how she used to attract birds to her garden, so now I think to do something with her like with the nuts and the bird things, it’s something to do that breaks the monotony up of that day and she enjoys doing it. She’ll talk to me the next time she sees me …then she’ll talk to her son and daughter-in-law about it when they come, …and it’s letting them see, that her time here isn’t just about being cared for physically, and in a way you could almost say it was a spiritual kind of care really. Care worker

By including examples such as this in a final report to each home, some staff were able to identify that they felt that this valued what they did.

Similarly, in Study 2, encouraging participants to reflect on their situation, enabled them to gain new insights. For some, the simple process of taking part in the study helped them consider their situation in preparation for the interview. One such example came from a family carer who commented:

I think possibly with you coming I’ve been sitting thinking about things. I worry what will happen to her if anything happens to me.

For others, such awareness resulted from the processes associated with the member checks. For example, a family carer remembered some of the better times with her recently deceased husband through the feedback process:

Do you know, looking back at this, when he could add up – I can’t remember him managing to do those things like. It’s been so long since he’s managed to do that, that I can’t remember it Philip. Well, I can but it seems an awful long while … I think that’s marvellous, I do. I had forgotten I’d done that, it brings it all back to me – it really does. Could I have a copy of those?

The key to promoting an enhanced awareness of the position of self is for the researcher to ensure that all conversations are dialectical (Rodwell 1998). This means that the constructions of the participants should be subject to the challenge of opposing views so that the individual is able to either justify their constructions in the face of the challenge or amend them in light of the new information. The hermeneutic dialectic process in the community study offered participants the opportunity to be exposed to the perspectives of other members of their stakeholder groups. While none of the participants demonstrated that they gained an enhanced understanding from this, using questions such as the one below encouraged them to consider such perspectives:

People have identified some things … that they value about their carers … and I would just like you both to see if they … apply to (you) at all …

Such exposure to divergent constructions can be both educative and empowering because this process offers participants the opportunity to enrich their own constructions (Erlandson *et al.* 1993). While it is difficult to conclude with confidence that participants gained an enhanced awareness of their situation, there are occasions in these two studies when this appears to be what has happened.

### An enhanced awareness of the position of others

An enhanced awareness of the position of others reflects the expectation that, as a result of being involved in constructivist research, individual participants should have an improved understanding of the constructions of others from different stakeholder groups (Guba & Lincoln 1989). It may be that the individuals never reach agreement with these different perspectives. However, they should have some additional understanding about the causes of these differing opinions, together with a degree of respect for them (Rodwell 1998).

In one example, in Study 2, the member checking process encouraged a family carer to reconsider his views about his wife. When asked to read the quotations in relation to the emerging conceptual framework, noting that he had concerns on how he became agitated when he wanted his wife to do things that he believed she could do; he commented:

FC To a certain extent that’s right but it’s not always the case. Perhaps I am a little misunderstanding with what she’s got and don’t appreciate it that she can’t do it. I mean I can’t be inside that person.

Observation of exchanges such as this in the hermeneutic process suggests an enhanced awareness of others.

Sharing views between stakeholder groups in Study 1 posed different difficulties when considering the confidentiality of participants, such as the risk that participants could be identified through the examples of care that they discussed. Opportunities to support different stakeholder groups in developing enhanced understanding of others were created in interviews. In the example below, the interviewer was able to present the views of some residents to a member of care staff:

They (residents) have said to me that it’s boring in the afternoon..and they say that the afternoons are so long which is why watches become so important to them, they feel they need to be able to tell the time.

At times, when those being interviewed replied in the affirmative, it was difficult to judge how much this was the development of an enhanced awareness of others, or simply a polite response.

The final report for each home in Study 1 was another vehicle used to support an enhanced understanding of others. One staff member described how surprised she was to read about the different activities that family care givers engaged in during their visits, which gave her an enhanced awareness of the role of family members in the home. Although residents were also given the opportunity to comment on their contribution and the final report, none of their comments demonstrated an enhanced awareness of others.

### Encouraging action by proving a rationale or impetus for change

One of the major outcomes of involvement in constructivist research is that it should encourage action. However, many constructivist studies are terminated before it has been possible to determine the extent to which desirable actions have been identified and stimulated (Rodwell 1998).

Opportunities for participants to identify how their involvement in Study 1 encouraged action were given through interviews and informal conversations. For example, it was evident in one care home that most of the paperwork was being carried out in the office, with limited opportunities to interact with residents. This observation had been shared through informal conversations with staff resulting in one nurse, describing how she had changed her practice, contributing towards more personal relationships:

Well I have changed the way I do things. I will go out now and sit outside the lifts to do my writing and the other night, we had a good communication time, I said ‘well lads it’s Saturday night, what are we going to do now?’ And Henry said well we usually have something fancy on a Saturday night and he was talking about cakes and things, so it was good.

The report to each care home had the potential to be used as a vehicle to stimulate action as it included suggestions for change from participants. After the report was circulated in each home, key participants were approached and asked what they felt could come from the research or how they might use the suggestions for improvement that had been included in the final report. These informal discussions could be described as a way of encouraging action.

In Study 2, some participants found that action was encouraged as a result of discussing aspects of their situation. This was reflected in the comments by a professional carer, who considered the impact on the family carer, since the daughter had left the family home – this left the family caregiver with less practical assistance and, as her husband had communication difficulties, this would mean that she would lack conversation at home:

Int But has the support increased?

FC No she only does the two evenings. I don’t know if she could apply for more vouchers through Social Services, I mean she could have had a day time sit, gone to the shops and we could have sat with Ian. I don’t know if she is thinking about anything like that. Perhaps I ought to mention it to her.

In both studies, participants were encouraged to consider action through the opportunity to discuss their situation with the researcher.

### Enabling action by providing the means to achieve change

In addition to being encouraged to act, it is expected that, as a result of involvement in a constructivist study, participants should be enabled and empowered to act (Erlandson *et al.* 1993). Moreover, with a time limited study, it may be difficult to establish the extent to which participants have been enabled to take action as a result of their involvement. Nolan *et al.* (2003) suggest that enabling action comes by providing the means to achieve or at least begin to achieve change. In Study 1, the report for each home became a vehicle to enable action with different participants across the three homes describing an intention to use the suggestions made in the report. In addition, for both studies, the extent to which they provided the means to achieve change is strongly influenced by the way in which the findings can be used to develop relevant recommendations for the development of future policy, practice, education and research.

## Discussion

Constructivist research provides a means of enabling the inclusion of multiple perspectives in the research process (Rodwell 1998). An increasing emphasis on participatory research methods suggests that knowledge creation is no longer the exclusive domain of academics, but a more inclusive activity (Rolfe 2000) with the acknowledgment that different types of knowledge are of value (Park 2001). Furthermore, older people bring different views derived from lived experience that can provide new insights to projects (Mountain 2003). However, to support older people to participate in the creation of knowledge requires partnership working throughout the research process (Dewar 2005, Clarke *et al.* 2009). Hanson *et al.* (2006) present a case for using the AVS model matrix in enabling older people and their carers to be involved in all aspects of this process. Both of the studies reported in this paper were approached with an awareness and acceptance of the significance of the authenticity criteria. At the planning stages (undertaken in 2001–2002), there was limited guidance on how to design studies that used opportunities to demonstrate each aspect of the criteria. As a result, the authenticity criteria were used as a reference to guide decisions to involve older people and their carers in all aspects of the research process, rather than to plan strategies actively. Upon completion of each study, the AVS model matrix provided a useful structure in retrospectively assessing the quality of each study (Table 5). Practical strategies were identified during this process that reflected how the authenticity criteria might be used to promote the inclusion of the voices of frail older people and their carers in the generation of knowledge through constructivist inquiry. Following the AVS model (Hanson *et al.* 2006), the following sections identify how each criterion might be adopted in the planning of the research, during the research process and in the products of the research.

**Table 5:** Use of the AVS model matrix (after Nolan *et al.* 2003, Hanson *et al.* 2006)

	Planning	Process	Product
Equal access	Use of written information in different formats; Sampling methodology that enable voices of all stakeholders to be involved	Repeating what people said, accounting for sensory difficulties and adapting research design to take these into account	Providing summaries for all participants, going through their transcripts with them, to ensure all views are represented
Enhanced awareness of self	Awareness of examples that were identified in data collection that may enhance awareness of self or their stakeholder group	Introducing examples in interviews as part of the hermeneutic process, or through informal conversations during participant observation	Engaging participants in a discussion of the interview transcripts or report if one is produced
Enhanced awareness of others	Interview schedules developed as data analysis progresses to introduce divergent views from other stakeholder groups	Introducing divergent perspectives into the interview or through informal conversations in participant observation, being aware of issues of confidentiality and anonymity	Engaging participants in a discussion of the developing theoretical framework or the wider report if one is produced
Encouraging action	This does not become clear until the research is underway	Providing opportunities for participants to make suggestions if they identify issues that they feel warrant change	Engaging participants in a discussion of the theoretical framework or wider report
Enabling action	This does not become clear until the research is underway	Providing opportunities for participants to verbalize issues they may feel warrants change and encourage them to make suggestions for this to happen	Providing participants with an accessible product that reflects local issues that arose through the research process that they may wish to address on completion of the research

### Planning

In constructivist research, enabling access of all stakeholder groups is key to discovering the breadth of views available. Having a clear idea of how this is to be achieved through sampling is therefore crucial in the planning stage.

A number of approaches were used in the studies reported here. For example, providing accessible information about the study to all potential participants was one strategy that promoted equal access. A key factor in both studies was being honest with older people about their involvement in the research and what was likely to be achieved (Davies & Nolan 2003). In both studies, it was acknowledged that there would be challenges in accessing the views of older people because of increasing frailty (Higgins 1998).

Guba and Lincoln (1989) suggest that all groups should have the opportunity to participate from positions of equal power. This was particularly problematic in the care home setting as there is unlikely to be equal power between the stakeholder groups alongside reluctance on the part of the older person to criticize the person that they will be relying on for essential care (Mitchell & Koch 1997). These factors were taken into account when planning the interviews for each group of participants. For example, interview schedules were developed as data analysis progressed to introduce divergent views from other stakeholder groups providing opportunities for participants to develop an enhanced awareness of self or others.

### Process

The studies reported in this paper suggest that physical and cognitive frailty in the care home or community has the potential to alter opportunities significantly for older people to be involved in the research. Mainly, this was due to speech difficulties, multi sensory impairment or problems with concentration on the part of the frailer participants. This raised the question that if older people lacked the physical capacity to make an equal contribution (in terms of the volume of their comments) there was a possibility that the contribution of those more able to withstand a longer interview might have been allowed to dominate. This was addressed through the prioritization of questions, using an intuitive judgement based on the relationship between the researcher and older person. This heightened awareness of the need for a flexible approach to each interview (Kayser-Jones & Koenig 1994), ensuring each older person had the opportunity to comment on the key emerging themes in the study while the more peripheral issues were left until the end and possibly not asked.

There were also situations where participants struggled to communicate and had to be asked to repeat what they had said due to the fact that so little had been understood. In this situation, there appeared to be a balance that needed to be achieved as constant requests to repeat the same phrase or idea might be perceived as being undermining to the participant yet, if they wish to express an idea that they believe to be relevant to the study, it seemed courteous to try to find out what they had said. While summarizing and offering feedback is a key role of the interviewer in constructivist research, in these situations it also helped in making the interview transcriptions as accurate as possible (Higgins 1998). For the participants whose speech was difficult to understand, a similar process of offering feedback was used. However, an additional aim with this feedback was to try to establish if any meaning had been lost as a result of misunderstanding certain words (Higgins 1998). Where the participant was accompanied by a family member for the interview, the assistance of the companion was enlisted when it was difficult to understand what the person was saying. However, confirmation of meaning was always sought from the participant (Bury & Holme 1990).

In each study, the hermeneutic dialectic process was enlisted through the use of interviews to facilitate the development of an understanding of the perspectives of members of other stakeholder groups. Rodwell (1998) argues from a social work context that as participants are invited to respond to the perspectives of other stakeholder groups, there should be elements of accommodation of these other viewpoints. As a result of this, the participant should be aware that they are expanding their constructions of their social context which enables them to improve their experience of the world around them (Erlandson *et al.* 1993). In each study presented in this paper, there was limited evidence to suggest that this was achieved. Although the researchers provided opportunities for each stakeholder group to comment on their and other’s perspective throughout the research process, very few participants took up these opportunities. However, if the involvement of older people in the evaluation of research is to be facilitated and sustained, then older people require their role to be clarified and any interpersonal issues they experience in groups to be acknowledged (Reed *et al.* 1999). While there is also the potential for raising awareness of the perspectives of others through the dissemination of findings, engaging with research participants to achieve enhanced understandings requires further work.

As the participants reach new and more sophisticated constructions which incorporate the perspectives of members of other stakeholder groups, the net result should be some form of action or decision making (Guba & Lincoln 1989). Providing participants with the opportunity to review their interview data was a particularly useful strategy in the community study where the researcher used questioning to support reflection on the insights gained. However, the degree to which the participants involved in these studies saw ‘action’ as part of their role in the research remains questionable. This raises an important issue in terms of context as other initiatives have found that older people and their carers wish research to be action oriented and experienced benefits in being involved in this process (Davies & Nolan 2003, Clarke *et al.* 2009). Strategies described in this paper may lead to future action on the completion of the study, although there is no way of substantiating if this might happen.

### Product

In both studies, summaries of interviews were given to all participants, with an opportunity to discuss these with the respective researchers. Engaging participants in a discussion about the interviews offered, an opportunity to explore the extent to which their views were represented, and enabled the researchers to present other viewpoints for discussion.

There were also opportunities in these discussions to engage participants in a discussion of the developing theoretical framework, although this was met with limited success. Nolan *et al.* (2003) emphasized the importance of the product of the research process being accessible to all stakeholders using ideas that are meaningful to them.

However, there is also the risk of rising expectations that might not be able to met through the research due to time limitations (Davies & Nolan 2003). In the care home-based study, the researcher gave each home a report based on the views of all stakeholder groups, engaging participants in a discussion of the report as it was being produced. This process resulted in an accessible product that reflected local issues. While this report exposed participants to both their own and others’ viewpoints, there was limited evidence that this had enhanced their awareness of themselves or others. The production of a report that represents the breadth of stakeholder views in this way may provide a focal point for shared decision making in the care environment.

## Conclusion

Involving older people and their carers in all aspects of the research process proved to be challenging, particularly in how each study was able to encourage or enable action. Nolan *et al.* (2003) suggests that, depending on the nature of the research, not all areas in the AVS model matrix need to be completed. However, the use of the AVS model matrix supported reflection at several stages of the research process, enabling the authors to explore ways of integrating quality criteria into the research process in an accessible format for older people, family caregivers and staff. Indeed, the AVS criteria has been used actively to promote user involvement at each point in the research process in the AVS centre (Hanson *et al.* 2006) and to evaluate peer education programme for advance care planning for older adults (Clarke *et al.* 2009). This suggests that for older people and carers to be full participants in the production of knowledge, opportunities for their involvement must be considered at a number of points throughout the research process. This paper suggests that there are differences in how the AVS model matrix may be applied depending on the context and subsequent design of the research and provides practical suggestions for the involvement of older people, families and staff in the research process.

**What is already known about this topic**

- Constructivist research values the active involvement of users and carers.
- The Authenticity criteria provides a framework for evaluating this involvement, although it has been criticized for its inaccessible language.
- The AVS model has been developed to support planning for the engagement of users and carers in all stages of the research process, although the literature lacks suggestions for specific interventions.

**What this paper adds**

- The AVS model can be used to evaluate the involvement of older people, families and staff participants in the research process.
- Practical strategies that illustrate how the AVS model might be used to support the active involvements of frail older people and their carers in the research process are proposed.

**Implications for practice and/or policy**

- The authenticity criteria are useful to guide decisions to involve older people and their carers in all aspects of the research process.
- The use of the AVS model matrix supports the integration of quality criteria into the research process in an accessible format for older people, family caregivers and staff.

The use of the authenticity criteria to assess the quality of qualitative research is relatively new, and as such presents its own challenges in its application in the research process, with older people and their carers. As a relatively new methodology, it is important that researchers document their experiences, highlighting the challenges and the benefits of the different ways in which the authenticity criteria may be used in a way that promotes user and carer involvement in research.
